# Iron Catalysts with Silyl‐NHC Chelate Ligands: Controlling Factors for Hydroboration vs. Dehydrogenative Borylation of Olefins

**DOI:** 10.1002/asia.202500709

**Published:** 2025-07-21

**Authors:** Nozomu Ishiwata, Takashi Komuro, Kasumi Takahashi, Yasuaki Zenzai, Hiromi Tobita, Hisako Hashimoto

**Affiliations:** ^1^ Department of Chemistry, Graduate School of Science Tohoku University 6‐3 Aramaki Aza‐Aoba Aoba‐ku Sendai 980–8578 Japan

**Keywords:** Catalyst, Iron, Ligand design, Olefin borylation, Silyl complex

## Abstract

Various iron complexes **1**, featuring silyl‐NHC chelate ligands (NHC = N‐heterocyclic carbene) were synthesized and investigated for their catalytic performance in the borylation reactions of olefins. Complexes **1** facilitated both hydroboration and dehydrogenative borylation of olefins with HBpin, yielding borylalkanes and borylalkenes, respectively. The selectivity between the two pathways was found to be governed by the steric effect of a substituent on one nitrogen atom of the NHC moiety. Possible catalytic mechanisms including hydroborato and β‐agostic‐alkyl complexes as potential resting states or intermediates are proposed based on stoichiometric reactions.

## Graphical abstract

Silyl‐NHC chelate iron complexes with various substituents on NHC were synthesized and demonstrated to be active catalysts for hydroboration and dehydrogenative borylation of olefins. The selectivity of these borylation reactions is significantly controlled by the steric bulk of a substituent on the NHC nitrogen.

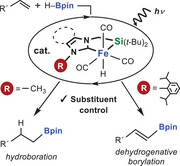

## 1 Introduction

**Figure 1:**
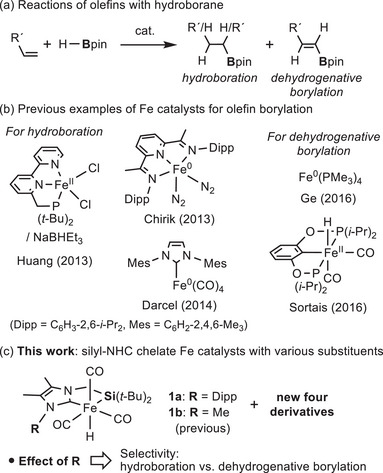
Iron catalysts for borylation reactions of olefins.

Borylation reactions of abundant olefins using metal catalysts are of great interest as straightforward methods to produce organoboron compounds, which are useful precursors of materials and pharmaceuticals.^[^ ^1^, ^2^ ^]^ Among such reactions, olefin hydroboration, which commonly occurs by treatment of olefins with hydroboranes to give borylalkanes is the most actively investigated (Figure 1a).^[^ ^1^, ^2^, ^3^ ^]^ On the other hand, dehydrogenative borylation of olefins, traditionally recognized as a major side reaction of hydroboration has recently garnered considerable attention as an attractive strategy for accessing borylalkenes.^[^ ^1^, ^2^, ^4^ ^]^ Since both types of products can be derived from the same reactants, control of the selectivity between these two pathways poses a significant challenge for practical synthesis.

Although precious metals are commonly used as catalysts for olefin borylation reactions, the use of less toxic and earth‐abundant iron in place of these metals is highly desirable from a sustainability perspective.^[^ ^2^, ^3^, ^4^ ^]^ To activate iron, which is relatively electron‐poor and less reactive compared to precious metals, electronically enriching the Fe center in complexes is likely to be a key factor in developing high‐performance catalysts. Thus, recent studies on olefin borylation reactions have incorporated low‐valent Fe centers and electron‐donating supporting ligands into catalyst design.^[^ ^3^, ^4^ ^]^ For instance, several iron complexes in Fe(0) or Fe(+II) oxidation state supported by pincer ligands (*P,N,N*‐type, *N,N,N*‐type, *P,B,P*‐type), bidentate chelates (*C,O*‐type, *P,N*‐type, *N,N*‐type), phosphines, as well as NHCs (N‐heterocyclic carbenes) have been developed as catalysts for olefin hydroboration (Figure 1b).^[^ ^3^ ^]^ Among these, two iron complexes, i.e., Fe(PMe_3_)_4_^[^ ^4a^ ^]^ and Fe(POCOP)(H)(CO)_2_ [POCOP = C_6_H_3_‐2,6‐{OP(*i*‐Pr)_2_}_2_],^[^ ^4b^ ^]^ have been used as catalysts for dehydrogenative borylation of olefins. These systems demonstrate high selectivity for either hydroboration or dehydrogenative borylation. However, the fundamental factors or origins governing such selectivity remain largely unclear.

Our group has also explored strategies to enhance the activity of iron complexes toward bond activation by employing supporting ligands with strong σ‐donation characteristics,^[^ ^5^ ^]^ such as silyl groups^[^ ^6^ ^]^ and NHC carbenes.^[^ ^7^ ^]^ Recently, we synthesized silyl‐NHC chelate iron complexes **1a** and **1b** (Figure 1c), which served as active catalysts for the double hydroboration of nitriles with pinacolborane via preactivation with photo‐irradiation.^[^ ^5a^ ^]^ In this study, we focused on the borylation reactions of olefins using **1a,b**, and their newly synthesized analogues.^[^ ^8^, ^9^ ^]^ These complexes also functioned as catalysts in the borylation reactions, with substituents exerting a significant influence on selectivity. Thus, a simple modification to the substituents of the NHC moiety profoundly affected selectivity, thereby directing the reaction pathways toward either hydroboration or dehydrogenative borylation. Herein, we report the synthesis and structural features of these catalysts along with deeper insights into selectivity in olefin borylation reactions. We also propose possible reaction mechanisms based on stoichiometric experiments.

## 2 Results and Discussion

### 2.1 Synthesis and Characterization of Silyl‐NHC Chelate Complexes of Iron

**Scheme 1:**
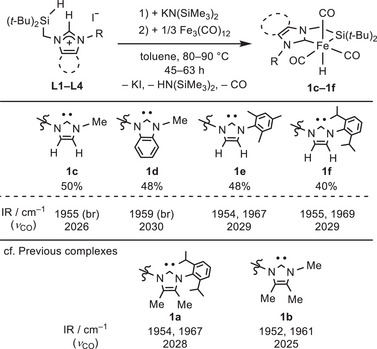
Synthesis of silyl‐NHC chelate iron complexes **1c–1f** and comparison of their IR data with **1a** and **1b**.

Treatment of *N*‐(hydrosilyl)methyl imidazolium salts **L1–L4** with the base KN(SiMe_3_)_2_ in toluene followed by the thermal reaction with Fe_3_(CO)_12_ at 80–90 °C afforded silyl‐NHC chelate complexes Fe(^SiC^L1–L4)(H)(CO)_3_ (**1c–1f**) in 40%–50% isolated yields (Scheme 1).

**Figure 2:**
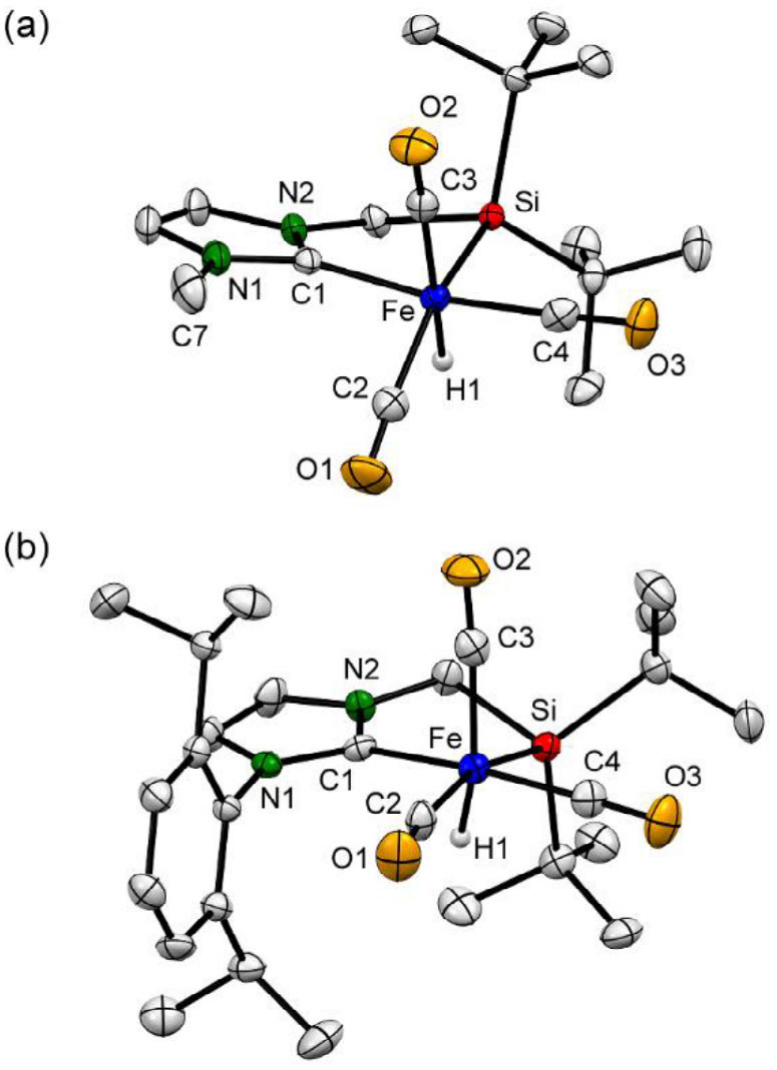
Crystal structures of (a) **1c** and (b) **1f**. Hydrogen atoms except hydrido hydrogens were omitted for clarity. Selected bond distances (Å) and angles (°) for **1c**: Fe─Si = 2.3565(6), Fe─C1 = 1.9849(19), Fe─C2 = 1.802(2), Fe─C3 = 1.796(2), Fe─C4 = 1.769(2), Fe─H1 = 1.48(2); Si─Fe─C1 = 81.00(6), Si─Fe─C2 = 151.94(7), Si─Fe─C3 = 104.94(7), Si─Fe─C4 = 85.36(7), C1─Fe─C4 = 166.35(9), Si1─Fe─H1 = 66.0(9), C3─Fe─H1 = 169.8(9). For **1f**: Fe─Si = 2.3711(19), Fe─C1 = 1.986(6), Fe─C2 = 1.800(7), Fe─C3 = 1.796(8), Fe─C4 = 1.767(7); Si─Fe─C1 = 81.06(18), Si─Fe─C2 = 162.8(2), Si─Fe─C3 = 97.4(2), Si─Fe─C4 = 84.2(2), C1─Fe─C2 = 99.0(3), C1─Fe─C3 = 89.9(3), C1─Fe─C4 = 163.3(3), C2─Fe─C3 = 99.8(3), C2─Fe─C4 = 92.8(3), C3─Fe─C4 = 99.8(3).

All complexes **1c–1f** were sufficiently characterized by multiple spectroscopies, and the structures of **1c**, **1d**, and **1f** were further confirmed by single crystal X‐ray diffraction (SC‐XRD) analysis [see the Supporting Information]. The SC‐XRD structures are depicted in Figure 2 (a) for **1c** and (b) for **1f**, and Figure S1 for **1d**.^[^ ^10^ ^]^ Crystal structures of the three complexes adopt a distorted octahedral geometry containing a bidentate silyl‐NHC chelate ligand. The silyl group, the NHC moiety, and the hydrido ligand adopt mutually *cis* positions in all cases; They are all strong *trans*‐influence groups. In comparison between **1c** and **1f**, the Dipp group on the NHC moiety in **1f** is significantly bulkier than the Me group in **1c** and sterically shields the coordination sphere around the metal center. In the ^1^H NMR spectra, the presence of Fe─H in **1c–1f** is confirmed by the signals observed in the characteristic metal‐hydride region (ca. −9 ppm).^[^ ^11^ ^]^ The ^29^Si{^1^H} NMR spectra of **1c–1f** exhibit a signal at ca. 79 ppm, characteristic of metal‐bound silicon. The IR spectra of **1c–1f** in toluene displayed two or three absorption peaks for CO stretching vibrations in the region of 1954–2030 cm^−1^ (Scheme 1). These values are close to those observed for analogous complexes **1a** (1954, 1967, and 2028 cm^−1^) and **1b** (1952, 1961, and 2025 cm^−1^).^[^ ^5a^ ^]^ This suggests that the electron‐donating ability of the silyl‐NHC ligands in **1a–1f** is approximately comparable, regardless of the substituents on the NHC units.

**Figure 3:**
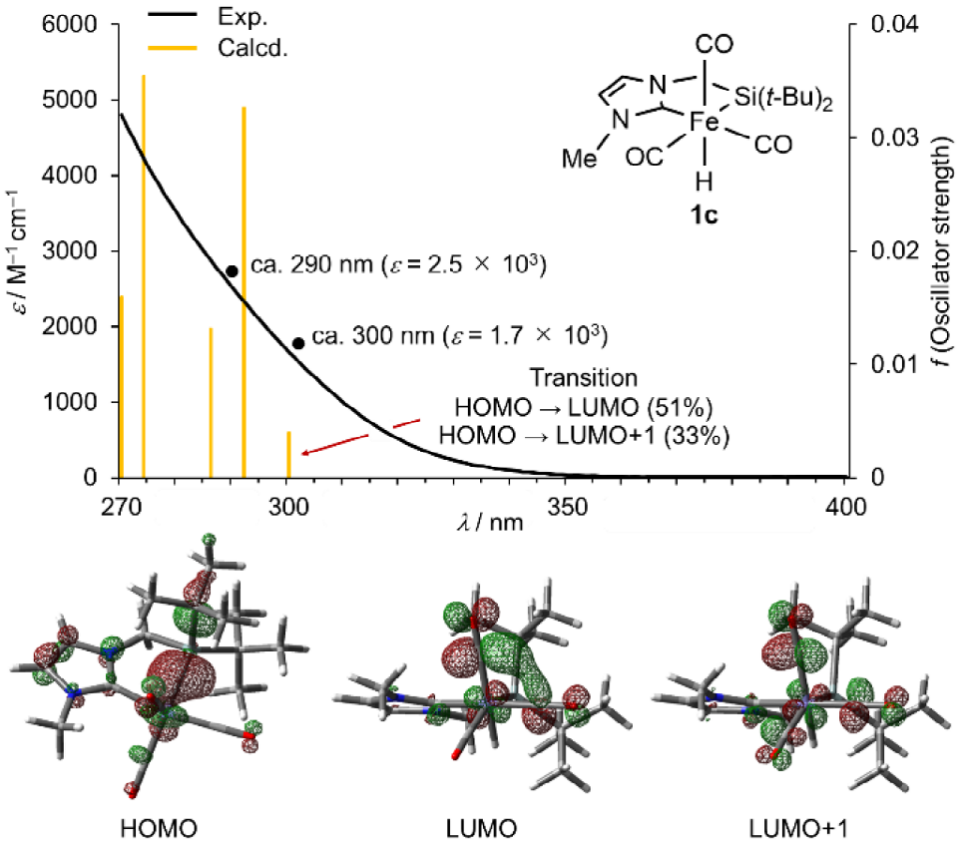
UV–vis spectrum of complex **1c** (black line), transitions based on TD‐DFT calculations of **1c‐opt** (orange bars), and important Kohn‐Sham orbitals (HOMO, LUMO, and LUMO+1) of **1c‐opt** (isovalue = 0.05).

As mentioned above, complexes **1a,1b** acted as catalysts under photo‐irradiation. Thus, the light absorption properties of the newly synthesized complexes **1c–1f** were also investigated. Consequently, all complexes exhibited similar UV‐vis spectra (see Supporting Information). The UV‐vis spectrum of **1c** in THF presenting in Figure 3 as a representative example, exhibits broad absorption extending to 350 nm. TD‐DFT calculations suggest that the lowest energy absorption appears at 300 nm, primarily attributed to the HOMO–LUMO transition with a substantial contribution from the HOMO–LUMO+1 transition (Figure S2 and Table S6). HOMO is mainly composed of an Fe─Si σ‐bonding orbital, while LUMO and LUMO+1 are mainly composed of Fe(d)─CO π*‐antibonding orbitals (Figures 3 and S3). Therefore, photo‐absorption around 300 nm is expected to induce dissociation of a CO ligand from **1c** generating coordinatively‐unsaturated active species.^[^ ^12^ ^]^

### 2.2 Catalytic Borylation Reactions of Styrene Using Silyl‐NHC Iron Complexes with Various Substituents on NHC

We initially examined catalytic activity of silyl‐NHC iron complexes **1a–1f** in the reaction of styrene with pinacol borane (HBpin) under photo‐irradiation and compared product yields. Thus, a solution of styrene and HBpin containing 5 mol% of complexes **1a–1f** was irradiated with light (*λ* > 300 nm), yielding a mixture containing hydroboration products **2a‐** *l* (linear), **2a‐** *b* (branched), and dehydrogenative borylation product **3a** together with hydrogenation product **4a** (Table 1). When complexes **1b**, **1c**, and **1d**, featuring a methyl substituent on the NHC moiety, were used (Entries 2–4), borylalkane **2a** was predominantly formed as the main product via hydroboration. This result also indicates that the imidazole and benzimidazole backbones of the NHC moiety had minimal effect on the selectively of the borylation products. In addition, linear (*anti*‐Markovnikov) hydroboration product **2a‐** *l* was preferably formed in comparison with branched (Markovnikov) product **2a‐** *b* (**2a‐** *l*:**2a‐** *b* = 1:0.1–0.21) in these cases. Among them, the use of catalyst **1c** resulted in the best selectivity of linear product **2a‐** *l* (Entry 3). In contrast, the use of complexes **1a**, **1e**, and **1f**, which feature bulky substituents on the NHC moiety, resulted in dehydrogenative borylation to yield **3a**, along with hydrogenation to form **4a** as main products (Entries 1, 5, and 6). Thus, the selectivity was significantly influenced by the bulkiness of the substituent R of the NHC moiety, which is positioned near a CO ligand on Fe, trans to the silyl group. Among these, catalyst **1a** demonstrated the best performance (Entry 1). Additionally, as part of control experiments, the reactions using **1a** and **1c** without photo‐irradiation were examined, yielding very little amounts of the products (Entries 7 and 8). This result indicates that photo‐irradiation is also indispensable for the progression of this reaction.

**Table 1:** Borylation reactions of styrene with HBpin catalyzed by silyl‐NHC chelate iron complexes.^a)^

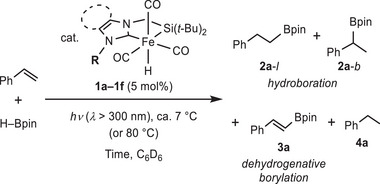					
Entry	Cat. (R)	Time / h	NMR yield / %		
			2a‐*l*/2a‐*b*	3a	4a
1	**1a** (Dipp)	4	9 (9/< 1)	45	38
2	**1b** (Me)	2	82 (68/14)	4	13
3	**1c** (Me)	2	56 (51/5)	12	17
4	**1d** (Me)	4	54 (47/7)	11	17
5	**1e** (Mes)	3	18 (18/< 1)	32	37
6	**1f** (Dipp)	2.5	18 (18/< 1)	40	39
7^b)^	**1a** (Dipp)	100	< 1 (< 1/0)	1	< 1
8^b)^	**1c** (Me)	100	9 (9/0)	4	5

^a)^ Reaction conditions: olefin (93 µmol), HBpin (94 µmol), catalyst (4.7 µmol, 5 mol%), and C_6_D_6_ (0.5 mL).

^b)^ The reaction was performed by heating at 80 °C without photo‐irradiation.

### 2.3 Scope of Olefins in the Borylation Reactions

Encouraged by these results in Table 1, we investigated the scope of mono‐substituted alkenes, cycloalkene, and 1,1‐disubstituted alkenes in the borylation reactions, using **1c** and **1a** as representative catalysts (Table 2). Complex **1c** was selected rather than **1b** because of its higher linear‐product selectivity for the styrene hydroboration (Table 1). When catalyst **1c** was used, both arylalkenes and alkylalkenes were preferentially hydroborated to give borylalkanes **2** in 49%–95% NMR yields (Entries 1–7). The reaction rates were comparable to that observed in the styrene case. In these reactions, linear hydroboration products (**2‐** *l*) were also predominantly formed over branched ones (**2**‐*b*). The selectivity of hydroboration (**2**) over dehydrogenative borylation (**3**) tends to be higher when arylalkenes with electron‐donating *para*‐substituents are used [**2**:**3** = 21:1 (*p*‐Me) and 15:1 (*p*‐OMe) vs 4.7:1 (*p*‐H) and 11:1 (*p*‐F)]. On the other hand, reactions with arylalkenes using catalyst **1a** predominantly afforded dehydrogenative borylation product **3** over hydroboration products **2** in moderate yields (30%–43%) (Entries 10 and 12) [dehydrogenative borylation:hydroboration = 1.8:1 (for *p*‐Me) and 3.1:1 (for *p*‐F)]. The reaction rate for 4‐fluorostyrene with the electron‐withdrawing F group is faster than that of electron‐donating Me group. In the reaction of (*p*‐methoxy)styrene (Entry 11), the product yields were significantly low, and there was considerable formation of side products.^[^ ^13^ ^]^ Notably, regardless of the catalyst, the reaction using sterically hindered *tert*‐butylethylene led to hydroboration to give borylalkane **2** in 63%–95% yields (Entries 4 and 13, vide infra). The same reaction employing a lower catalyst loading (1 mol%) of **1c** produced borylalkane **2** in 87% yield (TON = 87) with high selectively (Entry 5). In the reactions of less hindered 1‐hexene and vinylcyclohexane using **1a** (Entries 14 and 15), competitive olefin isomerization predominantly occurred to give unreactive internal olefins and thereby significantly reducing the yields of products **2–4**. The catalytic reaction using **1c** was also feasible for two examples of disubstituted olefins, α‐methylstyrene and cyclohexene, affording borylalkanes **2** between 71% and 76% yields, respectively (Entries 8 and 9). In contrast, the same reaction using **1a**, which bears a bulky Dipp group, proceeded more slowly, resulting in lower yields (Entries 16 and 17). This can be attributed to excessive steric hinderance with the disubstituted olefins.

**Table 2:** Scope of olefins in the borylation reactions catalyzed by complexes **1a** and **1c**.^a)^

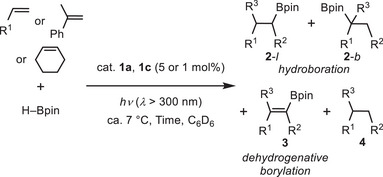						
Entry	R^1^ / R^2^ / R^3^	Cat.	Time / h	NMR yield / %		
				2‐*l*/2‐*b*	3	4
1	*p*‐MeC_6_H_4_ / H / H	**1c**	2	63 (52/11)	3	10
2	*p*‐(OMe)C_6_H_4_ / H / H	**1c**	2	59 (46/13)	4	11
3	*p*‐FC_6_H_4_ / H / H	**1c**	2	57 (49/8)	5	10
4	*t*‐Bu / H / H	**1c**	2	95 (95/0)	< 1	< 1
5^b)^	*t*‐Bu / H / H	**1c**	4	87 (87/0)	3	9
6^c)^	*n*‐Bu / H / H	**1c**	1	57 (57/0)	9	11
7^d)^	Cy / H / H	**1c**	2	49 (49/0)	1	3
8^e)^	Ph / H / Me	**1c**	10	71 (71/0)	4^f)^	8
9	–(CH_2_)_4_– / H	**1c**	10	76	2	20
10	*p*‐MeC_6_H_4_ / H / H	**1a**	10	17 (17/0)	30	26
11^g)^	*p*‐(OMe)C_6_H_4_ / H / H	**1a**	10	0 (0/0)	3	7
12	*p*‐FC_6_H_4_ / H / H	**1a**	2	14 (14/0)	43	39
13	*t*‐Bu / H / H	**1a**	4	63 (63/0)	5	8
14^h)^	*n*‐Bu / H / H	**1a**	1	11 (11/0)	2	5
15^i)^	Cy / H / H	**1a**	2	10 (10/0)	1	3
16^j)^	Ph / H / Me	**1a**	12	5 (5/0)	2^f)^	10
17^k)^	–(CH_2_)_4_– / H	**1a**	10	20	5	19

^a)^ Reaction conditions for Entries 1–4 and 6–17: olefin (93 µmol), HBpin (94 µmol), catalyst (4.7 µmol, 5 mol%), and C_6_D_6_ (0.5 mL); for Entry 5: olefin (0.48 mmol), HBpin (0.52 mmol), catalyst (4.7 µmol, 1 mol%), and C_6_D_6_ (0.5 mL).

^b)^ 1 mol% of **1c** was used.

^c)^ Isomerization products, 2‐hexene and 3‐hexene, were also formed as side products (total yield: 12%).

^d)^ Isomerization products, ethylidenecyclohexane and 1‐ethylcyclohexene, were also formed as side products in 6% and 24% yields, respectively.

^e)^ Conversions of α‐methylstyrene and HBpin: 93% and 85%, respectively.

^f)^ The (*Z*)‐isomer of dehydrogenative borylation product **3** was not formed.

^g)^ Conversions of (*p*‐methoxy)styrene and HBpin: 84% and 11%, respectively. GC‐MS of the reaction mixture: *m*/*z* 268 (dimer of the olefin).

^h)^ Isomerization products, 2‐hexene and 3‐hexene, were also formed as side products (total yield: 75%).

^i)^ Isomerization products, ethylidenecyclohexane (47% yield) and 1‐ethylcyclohexene (47% yield), were also formed as side products.

^j)^ Conversions of α‐methylstyrene and HBpin: 59% and 15%, respectively.

^k)^ Conversions of cyclohexene and HBpin: 53% and 60%, respectively.

### 2.4 Stoichiometric Reactions of Complex **1c** with HBpin and *tert*‐Butylethylene

**Scheme 2:**
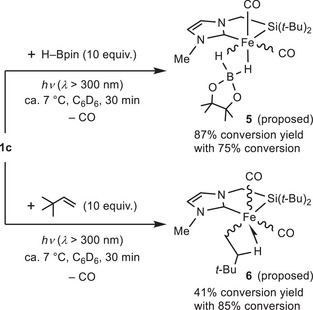
Stoichiometric reactions of complex **1c** with HBpin and *t*‐BuCH═CH_2_.

To gain insights into the mechanisms for this catalytic system, we also examined stoichiometric reactions (Scheme 2). Initially, the reaction of **1c** with HBpin (10 equiv.) was tested under photo‐irradiation conditions (*λ* > 300 nm) at ca. 7 °C. The ^1^H NMR spectrum measured after 30 min indicated the formation of hydroborato complex **5** as the main product in 65% NMR yield (87% conversion yield with 75% conversion of **1c**). Complex **5** was characterized based on NMR and mass spectroscopy (see Supporting Information). In the ^1^H NMR spectrum, two broad signals appearing between −11.0 and −7.6 ppm are assigned to the Fe─H─B bridging hydrogens, which are coordinated to Fe in a κ^2^‐fashion (Figure S50). The broadening of these signals is indicative of their attachment to B. Moreover, measurement of the ^1^H{^11^B} NMR spectrum under boron decoupling led to the change of these signals into sharp doublets (Figure S52), which is consistent with the presence of a direct B─H interaction. The ^11^B{^1^H} NMR spectrum shows a signal assignable to the hydroborato (H_2_Bpin) ligand of **5** at 35.3 ppm (Figure S54), which is close to the chemical shifts reported for iron complexes with a κ^2^‐H_2_Bpin ligand.^[^ ^3^, ^14^ ^]^ Additionally, a ^29^Si{^1^H} NMR signal was observed at 87.5 ppm, indicating that the Fe─Si bond is preserved in **5** (Figure S55). All these data strongly support the proposed structure of **5**, as illustrated in Scheme 2, despite the fact that isolating **5** was impossible due to its extreme instability. Notably, the NMR signals of **5** were occasionally observed during the final stage of the catalytic reaction, when the olefin had been completely consumed and a slight excess of HBpin remained. This observation may indicate that **5** serves as a resting state.

The reaction of **1c** with *tert*‐butylethylene (10 equiv.) was also examined, affording β‐agostic alkyl complex **6** as the main product in 41% conversion yield with 85% conversion of **1c** after 30 min. The ^1^H NMR spectrum exhibited the signals assignable to the alkyl ─CH_2_CH_2_(*t*‐Bu) moiety, along with the silyl‐NHC ligands (Figures S56–S58). The signal appearing in the metal–hydride region (at −7.90 ppm) as a doublet of doublet of doublets is assigned to one of the four methylene protons of the ─CH_2_CH_2_(*t*‐Bu) moiety, suggesting a β‐agostic interaction in **6**. This upfield‐shifted signal resembles those of previously reported β‐agostic alkyl complexes.^[^ ^15^ ^]^ Moreover, the ^29^Si NMR chemical shift of **6**, which was determined to be 83 ppm based on the ^1^H─^29^Si HMBC spectrum (Figure S61), is characteristic of a silyl complex. These NMR analyses strongly support the proposed structure of **6**, as illustrated in Scheme 2, while also considering the 18‐electron rule.

The formation of complexes **5** and **6**, each possessing two CO ligands, suggests the dissociation of a CO ligand from **1c** under photo‐irradiation. Moreover, the presence of the CH_2_CH_2_(*t*‐Bu) moiety in **6** indicates that migratory insertion of the olefin into the Fe─H bond occurred during the formation of **6**. Since alkyl complexes formed by such olefin insertion are regarded as key intermediates in iron‐catalyzed olefin hydroboration,^[^ ^2^, ^3^ ^]^ we suggest that complex **6** and/or its analogues also serve as intermediates in this system (vide infra).

### 2.5 Possible Mechanisms for the Catalytic Borylation Reactions Controlled by NHC Substituents

**Scheme 3:**
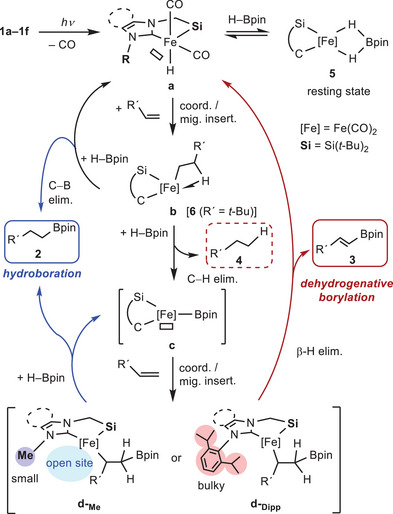
Proposed mechanisms for the hydroboration and dehydrogenative borylation of olefins catalyzed by complexes **1**. For the hydroboration, only the formation of linear products **2‐** *l* is selected as a representative example.

Based on the above‐mentioned experimental results, we propose possible mechanisms for the catalytic borylation reactions of olefins, as illustrated in Scheme 3. The catalytic reaction starts from the formation of coordinatively unsaturated iron complex **a** as an active species via photo‐irradiation, supported by the analysis of the UV‐vis spectrum and TD‐DFT calculations of **1c**. Olefin coordination to **a**, followed by migratory insertion of the olefin into the Fe─H, affords β‐agostic alkyl complex **b**, which corresponds to **6** observed in the stoichiometric reaction (Scheme 2). During the insertion step, a branched‐type alkyl complex also forms as a minor product, although it is not included in Scheme 3 for clarity. Intermediate **b** then reacts with HBpin to yield hydroboration product **2**, facilitating C─B bond formation, via B─H oxidative addition/C─B reductive elimination sequence or a σ‐complex assisted metathesis (σ‐CAM) mechanism,^[^ ^16^ ^]^ thereby regenerating complex **a**. The alternative pathway from **b** gives rise to boryl complex **c** with releasing hydrogenation product **4** via addition of HBpin and subsequent C─H reductive elimination. When the substituent (R^1^) on the olefin is a bulky *t*‐Bu group, the C─B reductive elimination predominantly occurs over the C─H reductive elimination after addition of HBpin, yielding **2**, with effectively reducing the steric congestion around the Fe center. Boryl complex **c** is then coordinated by an olefin, followed by the migratory insertion of the olefin to the Fe─B bond, giving intermediate **d**. From intermediate **d**, the reaction pathway is controlled by the steric hinderance of the substituent R on nitrogen of the NHC moiety. In the case of **d** with a small methyl substituent (**d‐_Me_**), the coordination site on Fe provides a sufficient space to allow for the reaction with HBpin, facilitating the formation of hydroboration product **2** along with the regeneration of complex **c**. Conversely, for **d** with a bulkier Dipp substituent (**d‐_Dipp_**), steric congestion at the Fe center inhibits the approach of HBpin. As a result, an alternative β‐hydride elimination occurs at the metal center, leading to the formation of dehydrogenative borylation product **3** along with the regeneration of starting active species **a**.^[^ ^3^, ^4^ ^]^ In addition, borato complex **5**, observed in the stoichiometric reaction of **1c** with HBpin, can be formed as a resting state through the reversible reaction of **a** in the presence of excess HBpin.

**Figure 4:**
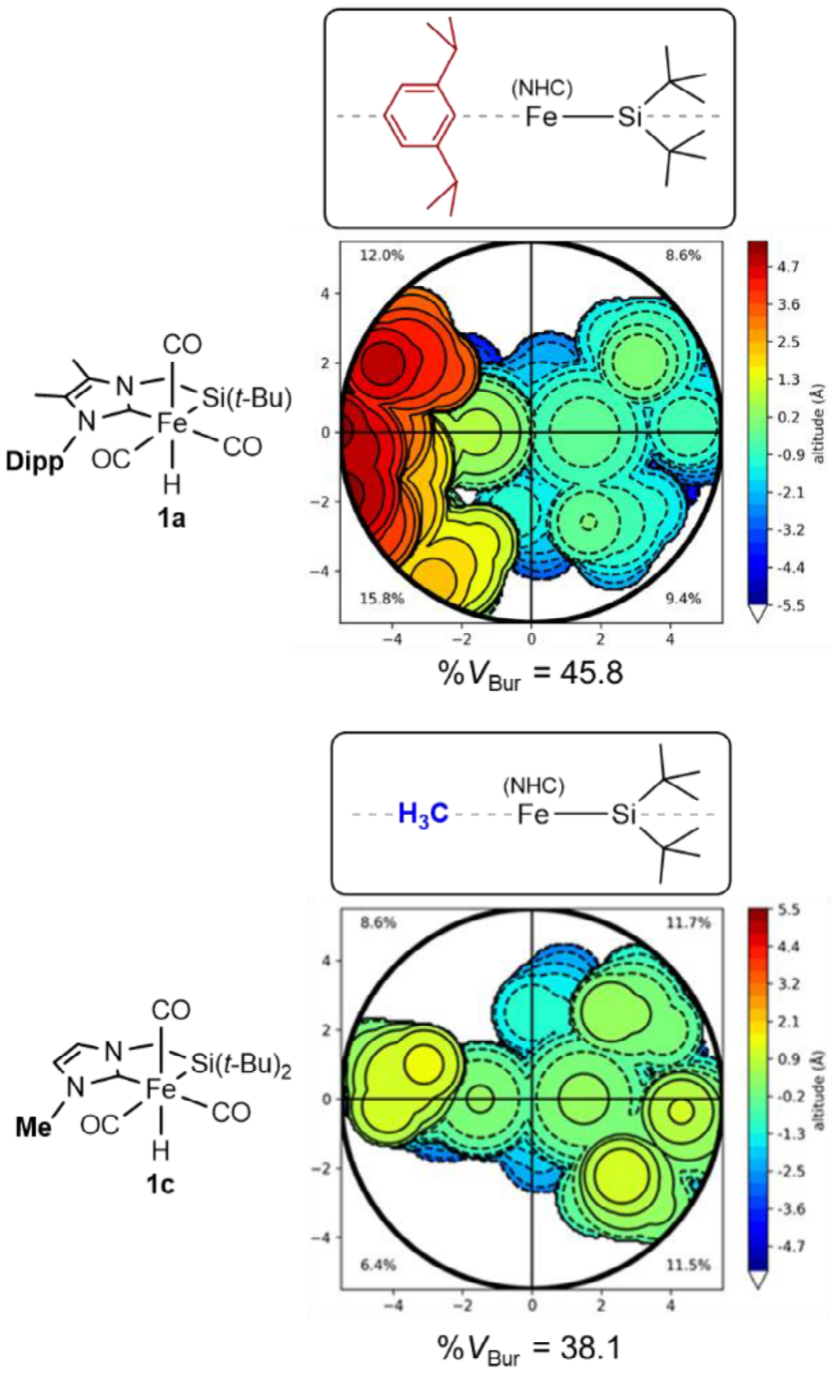
Analysis of steric maps around the metal centers of **1a** and **1c** and their buried volumes %*V*_Bur_ at the sphere radius (*r*) of 5.5 Å from the Fe center calculated by use of the SEQCROW bundle for the UCSF ChimeraX program (see Supporting Information). The CO and hydrido ligands were not included in the %*V*_Bur_ calculation.

Furthermore, to obtain more quantitative view of the steric hindrance of the silyl‐NHC ligands, we also calculated the buried volume %*V*_Bur_,^[^ ^17^ ^]^ based on the SC‐XRD structures using the SEQCROW bundle^[^ ^18^ ^]^ for the UCSF ChimeraX program.^[^ ^19^ ^]^ The buried volumes and corresponding steric maps of the silyl‐NHC chelate ligands in complexes **1a** (Dipp) and **1c** (Me) are illustrated in Figure 4 (see also Supporting Information). The %*V*_Bur_ values at the sphere radius (*r*) of 5.5 Å from the metal center were calculated to be 45.8% (**1a**) and 38.1% (**1c**). The comparison between these values clearly shows that the Dipp‐substituted silyl‐NHC ligand occupies a much larger space in the outer coordination sphere (see also Figure S5 for the change of %*V*_Bur_ in **1a** and **1c** depending on the sphere radii *r* of 2.0–9.0 Å). Thus, in the case of the reaction using **1a** with a buky substituent, steric repulsion makes access of HBpin to the iron center of intermediate **d** difficult, leading to favorable β‐hydrogen elimination to yield **3**. The present study clearly demonstrates that the steric bulkiness of the nitrogen substituent in the NHC moiety can fine‐tune the selectivity of catalytic borylation reactions of olefins.

## 3 Conclusions

We demonstrated that iron silyl‐NHC chelate complexes with different NHC moieties function as catalysts for both hydroboration and dehydrogenative borylation reactions of olefins. The strong σ‐donating ability of the silyl‐NHC ligands renders the Fe center electron‐rich, potentially facilitating the smooth progression of B─H activation of HBpin and β‐hydrogen elimination via C─H activation in the key steps for these reactions. Notably, the selectivity of these reactions is largely controlled by a simple modification of the bulkiness of the substituent on one nitrogen atom in the NHC. Catalysts **1b–1d**, featuring a small Me substituent on the NHC nitrogen, show a preference for hydroboration, whereas catalysts **1a,1e,1f**, bearing a sterically hindered Dipp or Mes substituent on the NHC nitrogen, enhance the selectivity for dehydrogenative borylation. Further applications of **1a–1f** as catalysts for other borylation reactions are under investigation.

## Conflict of Interests

The authors declare no conflict of interest.

## Supporting information



Supporting Information

## Data Availability

The data that support the findings of this study are available in the supplementary material of this article.
